# Evaluating the Effects of a Mobile Health App on Reducing Patient Care Needs and Improving Quality of Life After Oral Cancer Surgery: Quasiexperimental Study

**DOI:** 10.2196/18132

**Published:** 2020-07-27

**Authors:** Tze-Fang Wang, Rou-Chen Huang, Su-Chen Yang, Chyuan Chou, Lee-Chen Chen

**Affiliations:** 1 School of Nursing National Yang-Ming University Taipei City Taiwan; 2 Department of Nursing Far Eastern Memorial Hospital New Taipei City Taiwan; 3 Excellent Dental Center Taipei City Taiwan

**Keywords:** care needs, health information, mobile health app, oral cancer, technology acceptance, quality of life

## Abstract

**Background:**

Intervention with a mobile Health (mHealth) app can improve the efficacy of early detection of oral cancer and the outcomes for patients taking oral anticancer medications. The quality of life of oral cancer patients is significantly reduced within three months after surgery; also, their needs for nursing care and health information increase, mainly due to side effects and associated psychological problems.

**Objective:**

This study aimed to evaluate changes in the care needs and quality of life of patients with oral cancer after receiving the intervention of a newly developed mHealth app.

**Methods:**

After surgery, oral cancer patients were divided into an experimental group (n=50) who received the mHealth app intervention and a control group (n=50) who received routine health care and instruction. After 3 months of intervention, survey questionnaires were used to assess the patients’ quality of life, nursing care needs, and acceptance of the mHealth app.

**Results:**

The physiological care needs were significantly decreased in the experimental group compared with the control group (*P*<.05). Although the differences were not statistically significant, the psychological needs, communication needs, and care support needs all improved after the mHealth app intervention. The overall improvement in quality of life was higher in the experimental group than in the control group (–7.24 vs –4.36). In terms of intention to use, perceived usefulness, and perceived ease of use, the acceptability scores of the mHealth app were significantly increased after 3 months of intervention (*P*<.05).

**Conclusions:**

Compared with routine health care and instruction, for patients after surgery, the education/information intervention using the mHealth app significantly reduced their nursing care needs, improved their quality of life, and increased their acceptance of using an mHealth app on a mobile device. These findings can provide a theoretical basis for future health care app design and improvement. This study suggests that an mHealth app should be incorporated into the routine care of oral cancer patients to provide medical information quickly and improve their self-management abilities, thereby reducing the patients’ need for physiological care and improving their quality of life.

**Trial Registration:**

ClinicalTrials.gov NCT04049968; https://www.clinicaltrials.gov/ct2/show/NCT04049968

## Introduction

Oral cancer ranks sixth in incidence among cancers worldwide; among men, it ranks eighth in incidence rate (3.8/100,000) and mortality [1]. The World Health Organization (WHO) estimated that 657,000 new cases of oral cancer are diagnosed each year and that more than 330,000 people die annually from this disease [2]. Oral cancer is a serious issue in Taiwan; it is ranked fifth in number of cancer deaths and fourth among men in cancer morbidity and mortality. Indeed, the morbidity and mortality rates of oral cancer among men are almost 10 times those among women [3]. Although the five-year survival rate for oral cancer has increased to 70% to 80% [4,5], oral cancer patients still experience comorbidities, psychological distress, and reduced quality of life [6-8].

A prospective survey analyzed the quality of life of 83 oral cancer patients and found that physiological status (such as fatigue, pain, nausea, and vomiting symptoms) and physical activities (such as resuming sustainable work and leisure activities) significantly deteriorated within three months after surgery [9]. The patients’ social functioning, body image, and financial status also declined significantly after surgery. The incidence of anxiety/depression in patients with oral cancer after surgery reached 25% in patients surveyed within six months to six years [10]. Furthermore, pain, facial changes, and social activity were negatively correlated with psychological outcomes and quality of life. Even with the use of neoadjuvant treatments and observed improvement in the first year after surgery, the quality of life of patients within three months of surgery remained significantly reduced [11]. Therefore, there is still an urgent need for new approaches to improve the quality of life of patients with oral cancer after surgery.

The decline in the quality of life of patients after oral cancer treatment increases the demand for nursing care and health information, mainly due to the side effects and resulting psychological problems after treatment [12]. For example, compared with cancer patients who did not receive radiotherapy, cancer patients who received radiotherapy may report that their care needs and communication needs are not being met [13]. Similarly, cancer patients receiving chemotherapy may experience nausea, fatigue, decreased physical function, and emotional problems, all of which require care and attention [14]. Postoperative oral cancer patients have a series of nursing needs, including psychological needs, professional medical care needs, and particularly support needs in terms of interpersonal communication, including disease information, treatment options, and medication options, such as pain management [15-17]. However, most health-related information in manuals or books fails to meet these needs because the desired information may take too long to find and the level of writing may be too specialized for patients to understand. Instead, patients may want to quickly obtain specific information at any time and place [18]. When patients receive sufficient support to meet their needs, they can more effectively cope with their negative emotions and disease symptoms.

The popularity in many countries of smart devices such as mobile phones and tablets has led to increasing use of mobile health (mHealth) apps to quickly and efficiently transmit medical information and provide health care services to patients [19]. The WHO Global Observatory for eHealth defines mHealth as “medical and public health practice supported by mobile devices, such as mobile phones, patient monitoring devices, personal digital assistants (PDAs), and other wireless devices [20].” An mHealth app is a software program that runs on mobile phones, tablets, or other mobile devices (eg, smartwatches, wristbands) for health care and disease prevention [21]. In addition to monitoring health, an mHealth app can encourage healthy behaviors and provide patients with effective ways to manage disease [22,23]. Several mHealth apps have been developed for disease management approaches such as blood glucose control (diabetes) [24], hypertension control [25], depression treatment [26], remote cancer surveillance [27], and medication monitoring [28,29].

In this study, we developed an mHealth app for patients after oral cancer surgery. To the best of our knowledge, no study has investigated whether an intervention based on an mHealth app can affect the daily needs and quality of life of patients after oral cancer surgery. This study aimed to investigate whether the medical information and education provided through the developed mHealth app can reduce the care needs of patients and improve their quality of life after the intervention. We believe that patients and their families may benefit from this convenient physical and social support system, which enables patients to quickly obtain information to help them cope with their disease, reduce their anxiety, and improve their quality of life.

## Methods

### Study Design and Sample

This study used a quasiexperimental research design and convenience sampling. The study protocol was reviewed and approved by the internal review board of the Research Ethics Review Committee of Far Eastern Memorial Hospital (No. 105110-E). Study participants were recruited from the departments of Oral Surgery, Otolaryngology, Hematology Oncology, and Radiation Oncology at the Far Eastern Memorial Hospital in New Taipei City, Taiwan. The inclusion criteria were patients who were diagnosed with oral cancer by a physician and who underwent oral cancer surgery within one week; patients who were conscious and able to communicate in Mandarin or Taiwanese; and patients who agreed to participate in the study and possessed a smartphone. The exclusion criteria were patients who did not have oral cancer or patients who had oral cancer but underwent oral cancer surgery one week previously; patients who could not communicate in Mandarin or Taiwanese; patients who were unconscious or unable to answer the questions in the questionnaire; and patients who had cognitive impairment, dementia, or intellectual disability. After the well-trained researchers explained the study to the eligible participants who met the inclusion criteria, the participants who agreed and who provided signed informed consent were included in the study. Each patient was individually instructed by the investigator prior to discharge, and the content of the instruction focused on treatment messages, oral care, and social resource delivery. The intervention outcomes of the patients in both groups were surveyed after 3 months using questionnaires. The study was registered prior to launch (ClinicalTrials.gov NCT04049968).

### Data Collection and Features of the mHealth App

**Figure 1:**
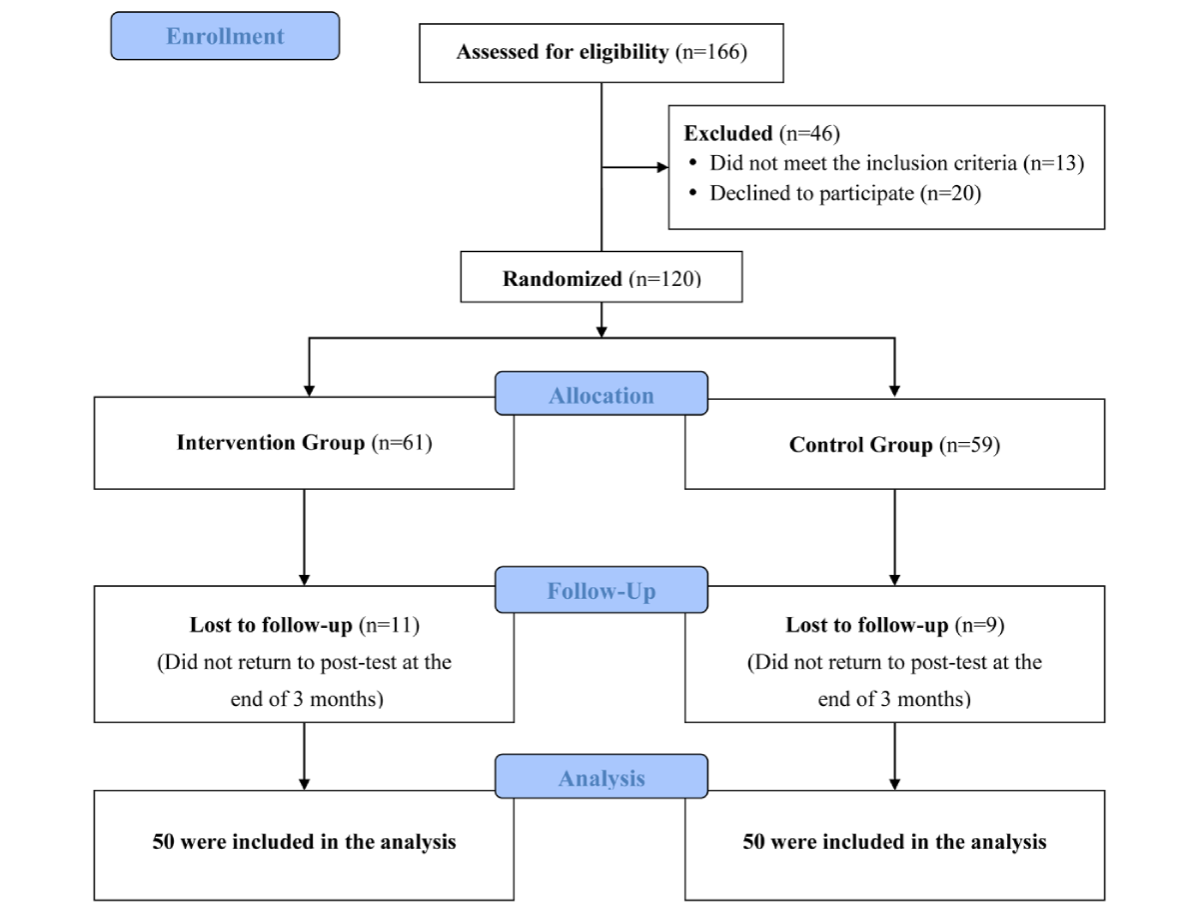
Flowchart of study sample selection.

All included patients were randomly divided into an experimental group and a control group (Figure 1). The patients in the control group received routine care and education, while the patients in the experimental group received 20 minutes of mHealth app education and guidance before being discharged from the hospital. The educational content included helping the participants to download, install, and use the mHealth app. It also included instructions to teach patients how to use the mHealth app at home after discharge to provide education about oral cancer treatment (surgical treatment, chemotherapy, radiotherapy, rehabilitation exercise) and included links to videos about self-recording of symptoms, available support groups, and other applicable information to help meet the needs of the patients. The two groups of patients received the same routine care before discharge. The patients in the experimental group and the control group returned to the hospital 3 months later to complete the questionnaires.

The mHealth app has four main interfaces: Latest News, Medical Information, Self-Recording, and Revisit Reminders. The Latest News interface provides the latest exhortations for patients after surgery and provides YouTube links to oral health education and head and neck rehabilitation videos. In addition, the interface contains a link to join to other patient groups through the LINE app. Therefore, patients with oral cancer after surgery can share information about their lives and treatment experiences after treatment. The Medical Information interface provides information about oral cancer, oral cancer treatment, pain information, hospice care, and any other supporting personnel or cancer treatment institutions. The Self-Recording interface enables patients to record their own postoperative information and symptoms, including date, body temperature, pain levels, oral ulcer, vomiting, skin reactions, and diarrhea. On their next return visit, patients can provide this information to their physician for reference and discuss the response of their disease to treatment. Finally, the Revisit Reminder interface provides a reminder function for patients to remember to return to the hospital. Screenshots of the interfaces are shown in Multimedia Appendix 1.

### Questionnaires

The questionnaires used in this study included a self-administered questionnaire containing questions about the demographic characteristics of the patients (age, gender, marital status, education level, financial status, and religion) and their clinical characteristics (cancer stage, surgical procedure, follow-up treatment, age at onset, and duration of illness); and a care needs scale, namely the short-form Cancer Needs Questionnaire (CNQ-SF), a 32-item self-administered questionnaire that evaluates 5 domains of patient needs, including psychological, health information, physical and daily living, patient care and support, and interpersonal communication needs. The CNQ-SF score ranges from 0 to 100, where 0 means no need and 100 means the highest need [15]. The questionnaires also included the European Organization for Research and Treatment of Cancer Quality of Life Questionnaire Core 30 (EORTC QLQ-C30) and Head and Neck Module (EORTC QLQ-H&N35) [30] and the Science and Technology Acceptance Model (TAM) scale, which was designed based on the information systems theory developed by Davis [31] in 1989 to evaluate patients’ responses to health information technology. The Cronbach α values of the care needs scale and quality of life scale were .94 to .77 and .92, respectively. The content validity index of the TAM scale was between 0.92 and 1.00, with an average of 0.96 (Cronbach α=.97). The questionnaires used in this study are shown in Multimedia Appendix 2.

### Statistical Analysis

Continuous variables are presented as mean (SD); categorical variables are presented as n (%). Differences in categorical variables were examined using the chi-square test or Fisher exact test, and the differences in the continuous variables were examined using the independent *t* test. The paired *t* test was used to examine the differences between measurements before and after the intervention. After adjusting for age and sex, multivariate linear regression was used to assess the associations between the patients’ care needs and quality of life before and after using the app. All statistical assessments were two-sided, and *P*<.05 was considered statistically significant. Statistical analyses were performed using SPSS version 22 for Windows (IBM Corp).

## Results

### Analytical Sample

A total of 120 participants were enrolled in this study. The experimental group included 61 postoperative patients who used the mHealth app within three months of discharge, while the control group included 59 postoperative patients who received routine care and instruction but did not use the app. After 3 months of follow-up, 11/61 patients in the experimental group (18%) and 9/59 patients in the control group (15%) did not return to the hospital to fill out the questionnaires. The final sample therefore included 50 patients in the experimental group and 50 patients in the control group (Figure 1).

### Background Characteristics of the Study Population

The study enrolled 100 postoperative oral cancer patients, including 92 men (92.0%) and 8 women (8.0%), with a mean age of 57.01 years (SD 8.87). Most patients were married (68/100, 68.0%), had a middle school education (37, 37.0%), were unemployed (61, 61.0%), earned less than 20,000 NT$ (US $677.13) per month (59, 59.0%), had a religious affiliation (87, 87.0%), and were self-caregivers (60, 60.0%). Most of the 100 patients were diagnosed with stage I (32, 32.0%) or stage II (32, 32.0%) cancer, without cancer metastasis (65, 65.0%), and the primary cancer was located at a buccal site in most cases (53, 53.0%). Of the 100 patients, 92 (92.0%) received tumor resection, 66 (66.0%) received radiation therapy, and 38 (38.0%) received chemotherapy. In follow-up treatment, 35/100 patients (35.0%) received radiation therapy and 3 patients (3.0%) received chemotherapy (Table 1).

**Table 1:** Baseline demographics and clinical characteristics of the patients in the study (N=100).

Variable			Total (N=100)	Experimental group (n=50)	Control group (n=50)		*P* value
**Demographics**							
	**Sex** **, n (%)**					.72	
		Male	92 (92.0)	47 (94.0)	45 (90.0)		
		Female	8 (8.0)	3 (6.0)	5 (10.0)		
	Age (years), mean (SD)		57.01 (8.87)	58.7 (7.56)	55.32 (9.79)		.06
	**Marital status, n (%)**					.39	
		Married	68 (68.0)	32 (64.0)	36 (72.0)		
		Other (unmarried/widowed/divorced)	32 (32.0)	18 (36.0)	14 (28.0)		
	**Education, n (%)**					.10	
		Below primary school	28 (28.0)	18 (36.0)	10 (20.0)		
		Middle school	37 (37.0)	19 (38.0)	18 (36.0)		
		Above high school	35 (35.0)	13 (26.0)	22 (44.0)		
	**Employment status, n (%)**					.54	
		No	61 (61.0)	32 (64.0)	29 (58.0)		
		Yes	39 (39.0)	18 (36.0)	21 (42.0)		
	**Income (NT$)^a^, n (%)**					.45	
		Less than 20,000	59 (59.0)	32 (64.0)	27 (54.0)		
		20,000-39,999	20 (20.0)	10 (20.0)	10 (20.0)		
		More than 40,000	21 (21.0)	8 (16.0)	13 (26.0)		
	**Religion, n (%)**					.77	
		No	13 (13.0)	6 (12.0)	7 (14.0)		
		Yes	87 (87.0)	44 (88.0)	43 (86.0)		
	**Primary caregiver, n (%)**					.41	
		Self	60 (60.0)	32 (64.0)	28 (56.0)		
		Other (spouse/child/caregiver)	40 (40.0)	18 (36.0)	22 (44.0)		
**Clinical characteristics, n (%)**							
	**Cancer stage**					.34	
		I	32 (32.0)	19 (38.0)	13 (26.0)		
		II	32 (32.0)	17 (34.0)	15 (30.0)		
		III	15 (15.0)	5 (10.0)	10 (20.0)		
		IV	21 (21.0)	9 (18.0)	12 (24.0)		
	**Tumor metastasis**					.06	
		No	65 (65.0)	28 (56.0)	37 (74.0)		
		Yes	35 (35.0)	22 (44.0)	13 (26.0)		
	**Primary site**						
		Lip	14 (14.0)	9 (18.0)	5 (10.0)		.25
		Buccal side	53 (53.0)	28 (56.0)	25 (50.0)		.55
		Hard palate	34 (34.0)	14 (28.0)	20 (40.0)		.21
		Posterior molar region	6 (6.0)	2 (4.0)	4 (8.0)		.68
		Tongue	44 (44.0)	23 (46.0)	21 (42.0)		.69
	**Previous treatment**						
		Tumor resection	92 (92.0)	48 (96.0)	44 (88.0)		.27
		Radiation therapy	66 (66.0)	33 (50.0)	33 (50.0)		>.99
		Chemotherapy	38 (38.0)	23 (46.0)	21 (42.0)		.68
	**Follow-up treatment**					.87	
		Radiation therapy	35 (35.0)	17 (34.0)	18 (36.0)		
		Chemotherapy	3 (3.0)	1 (2.0)	2 (4.0)		

^a^NT $1=US $0.034.

### The mHealth App Intervention Improved Quality of Life and Reduced Care Needs

Table 2 shows the statistical results of the patients’ scores on the global quality of life scale (EORTC QLQ-H&N35). The lower the score on the scale, the higher the patient’s satisfaction with their quality of life. At baseline, the total quality of life scores in the experimental group and control group were 32.15 and 28.99, respectively. After 3 months of intervention, the total quality of life scores in the experimental group and the control group were reduced to 24.91 and 24.63, respectively. Although the changes in the total scores between the two groups were statistically insignificant, the overall improvement in the intervention group was greater than that in the control group (–7.24 vs –4.36).

**Table 2:** Quality of life scores of patients in the study (N=100) before and after the mHealth app intervention measured with the European Organization for Research and Treatment of Cancer Quality of Life Questionnaire Core 30.

Variable			Before					After					
		Control group, mean (SD)		Experimental group, mean (SD)		*P* value^a^		Control group, mean (SD)		Experimental group, mean (SD)		*P* value	
Overall quality of life score			28.99 (16.40)		32.15 (18.65)		.37		24.63 (16.97)		24.91 (17.13)		.94
Change of overall quality of life score			N/A^b^		N/A		N/A		–4.36 (10.26)		–7.24 (12.77)		.22
**Quality of life scores**													
	Pain	19.50 (20.10)		22.50 (22.40)		.48		19.33 (18.24)		18.99 (20.89)		.93	
	Swallowing	25.50 (26.69)		31.50 (29.03)		.29		26.16 (24.80)		30.83 (26.09)		.36	
	Teeth	40.00 (34.99)		42.00 (37.38)		.78		38.66 (33.23)		40.00 (34.99)		.85	
	Opening mouth	41.33 (35.99)		48.00 (35.74)		.36		39.99 (31.58)		43.33 (33.16)		.61	
	Dry mouth	46.67 (33.67)		40.00 (36.89)		.35		45.33 (29.16)		34.66 (26.90)		.06	
	Sticky saliva	43.33 (35.16)		37.33 (35.41)		.40		39.99 (29.35)		23.33 (25.42)		.003^c^	
	Senses problems	18.33 (21.09)		18.33 (27.20)		>.99		15.66 (17.94)		14.66 (18.63)		.79	
	Coughing	17.33 (24.50)		24.00 (27.80)		.21		27.33 (94.44)		17.33 (24.50)		.47	
	Felt ill	16.67 (27.15)		21.33 (27.57)		.40		13.99 (27.01)		11.99 (21.03)		.68	
	Trouble with social eating	29.50 (28.78)		30.83 (27.63)		.81		25.66 (27.39)		29.66 (28.23)		.47	
	Speech problems	22.00 (21.59)		23.55 (28.36)		.76		18.22 (21.38)		16.22 (22.47)		.65	
	Trouble with social contact	16.27 (20.16)		15.33 (20.03)		.82		14.26 (20.47)		13.60 (18.75)		.87	
	Less sexuality	11.33 (18.27)		17.00 (24.86)		.20		7.66 (15.50)		12.66 (22.22)		.20	
	Pain killers	54.00 (50.35)		56.00 (50.14)		.84		40.00 (40.00)		44.00 (50.14)		.69	
	Nasogastric tube feeding	42.00 (49.86)		62.00 (49.03)		.046^c^		32.00 (47.12)		56.00 (50.14)		.015^c^	
	Nutritional supplements	28.00 (33.75)		45.00 (35.36)		.016^c^		23.00 (32.27)		35.00 (35.35)		.08	
	Weight loss	22.00 (41.85)		30.00 (46.29)		.37		6.00 (23.98)		6.00 (23.98)		>.99	
	Weight gain	28.00 (45.36)		14.00 (35.05)		.09		10.00 (30.30)		0		.024^c^	

^a^*P* value was used to identify statistical significance between the experimental group and control group.

^b^N/A: not applicable.

^c^*P*<.05.

The CNQ-SF measures baseline postoperative care needs (Table 3). The higher the scores on the scale, the higher the patient’s need for care. Before the intervention, the mean scores of the 5 care domains in the experimental group and control group were 26.33 vs 21.33 for physiological needs, 24.55 vs 26.27 for psychological needs, 13.50 vs 16.75 for communication needs, 26.92 vs 19.58 for care support needs, and 64.0 vs. 60.29 for health information needs, respectively. After 3 months of intervention, the mean scores for the experimental group and control group were 20.67 vs 20.25 for physiological needs, 18.18 vs 23.14 for psychological needs, 8.25 vs 12.75 for communication needs, 23.75 vs 17.67 for care support needs, and 63.86 vs 57.0 for health information needs, respectively. These results show that the experimental group (mHealth app intervention) had significantly reduced physiological needs compared to the control group (*P*=.015, Table 3). Although the results were not statistically significant, the experimental group had more obvious reductions in psychological needs, communication needs, and care support needs than the control group. Multivariate linear regression analysis also confirmed that after adjusting for age and sex variables, the experimental group had significantly greater improvement in physiological needs compared to the control group (*P*=.022, Table 4).

**Table 3:** Care needs of patients in the study (N=100) before and after the mHealth app intervention measured with the short-form Cancer Needs Questionnaire.

Variable	Before			After				Change		
	Control group, mean (SD)	Experimental group, mean (SD)	*P* value^a^	Control group, mean (SD)	Experimental group, mean (SD)	*P* value	Control group, mean (SD)		Experimental group, mean (SD)	*P* value
Physiological needs	21.33 (18.36)	26.33 (20.03)	.20	20.25 (15.95)	20.67 (15.45)	.90	–1.08 (7.80)		–5.67 (10.47)	.015^b^
Psychological needs	26.27 (23.07)	24.55 (23.98)	.71	23.14 (20.40)	18.18 (17.29)	.19	–3.14 (8.04)		–6.36 (13.88)	.16
Communication needs	16.75 (22.53)	13.50 (21.70)	.46	12.75 (21.94)	8.25 (17.97)	.27	–4.0 (12.74)		–5.25 (9.81)	.58
Care support needs	19.58 (21.33)	26.92 (18.52)	.07	17.67 (15.92)	23.75 (15.48)	.05	–2.0 (12.88)		–3.17 (7.79)	.59
Health information needs	60.29 (25.72)	64.00 (28.78)	.50	57.00 (25.95)	63.86 (23.61)	.17	–3.29 (19.06)		–0.14 (13.15)	.34

^a^*P* value was used to identify statistical significance between the experimental group and control group.

^b^*P*<.05.

**Table 4:** Multivariate linear regression analysis of patients’ care needs after the mHealth app intervention.

Model^a^			App use		
			β (95% CI)		*P* value
EORTC QLQ-H&N35^b^			–3.34 (–7.83 to 1.15)		.15
**CNQ-SF^c^**					
	Physiological needs	–4.24 (–7.88 to –0.60)		.022^d^	
	Psychological needs	–2.75 (–7.22 to 1.73)		.23	
	Communication needs	–0.99 (–5.49 to 3.51)		.67	
	Care support needs	–0.80 (–4.99 to 3.39)		.71	
	Health information needs	3.21 (–3.27 to 9.70)		.33	

^a^Model adjusted for sex and age.

^b^EORTC QLQ-H&N35: Organization for Research and Treatment of Cancer Quality of Life Questionnaire Core 30 and Head and Neck Module.

^c^CNQ-SF: short-form Cancer Needs Questionnaire.

^d^*P*<.05.

### Patient Acceptance of the mHealth App

Patient acceptance of the mHealth app was measured by intention to use, perceived usefulness, and perceived ease of use (TAM scale). At baseline, the mean scores in the experimental group for intention to use, perceived usefulness, and perceived ease of use were 2.54, 2.52, and 2.32, respectively. In the control group, the mean scores for the 3 aspects were 2.68, 2.49, and 2.49, respectively. After 3 months of the mHealth app intervention, the mean scores for the three aspects increased in the intervention group to 3.02, 2.95, and 3.01, respectively. All three aspects of app acceptability significantly increased after the intervention (*P*<.01, Table 5).

**Table 5:** Acceptability of the mHealth app by patients based on the Science and Technology Acceptance Model scale.

Variable	Before			After		
	Control group, mean (SD)	Experimental group, mean (SD)	*P* value^a^	Control group, mean (SD)	Experimental group, mean (SD)	*P* value^b^
Intention to use	2.68 (1.12)	2.54 (1.05)	.52	N/A^c^	3.02 (0.87)	.002
Perceived usefulness	2.49 (1.14)	2.52 (1.09)	.90	N/A	2.95 (0.99)	.004
Perceived ease of use	2.49 (1.04)	2.32 (0.77)	.37	N/A	3.01 (0.90)	<.001

^a^Independent *t* test with *P* value was used to identify statistical differences between the experimental group and the control group.

^b^Paired *t* test with *P* value was used to identify statistical differences before and after the mHealth app intervention.

^c^N/A: not applicable.

## Discussion

### Principal Findings

Previous studies showed that the physical function and oral function of oral cancer patients deteriorates significantly 3 months after surgery, accompanied by poorer body image and less social contact [9]. Birur et al [27] used a remote mHealth-based approach to establish an effective oral cancer screening program and found that the intervention could improve the efficiency of early detection of oral cancer. In addition, an intervention with a personalized mobile phone–based self-management app could improve outcomes for patients taking oral anticancer medications [28]. In particular, education and information received through mHealth apps has been shown to improve prevention and posttreatment outcomes in various clinical situations [19]. In addition to supporting these studies, this study further showed that compared with routine health care and instruction, the education/information intervention provided by the mHealth APP indeed reduced care needs; the study also showed that patients had a higher degree of acceptance of using mobile devices to learn about and manage their disease.

Quality of life scores have been linked to predicted survival of head and neck cancer patients [32]. Initiating supportive care as early as possible, with measures such as encouraging optimal nutritional intake and improving oral function by reducing symptoms, can help improve the quality of life of head and neck cancer patients after surgery [2]. In this study, although the results were not statistically significant, patients reported improved quality of life after the mHealth app intervention. We note that quality of life is a long-term state, and more than three months may be needed to observe statistically significant differences between control and intervention groups, as in other studies [33]. In this study, the intervention lasted only 12 weeks; thus, further investigation is needed to determine the ideal intervention time to measure the differences in outcomes.

Interestingly, most patients in the control group claimed that they had received medical care and sufficient information from the medical staff; however, more than half of these patients stated during revisits that they had forgotten or only remembered part of this information. This result may be partly due to the fact that the health education leaflets provided by the nursing staff are not easy to carry around or that the contents are relatively boring (patients’ statement), which may have reduced patients’ willingness to read the leaflets. In contrast, the mHealth app can enable patients to immediately access information on their medical condition, medications and dosages, or changes in symptoms whenever they want. It can also provide a useful reminder system to help patients manage their treatment schedules [33].

In general, patients with oral cancer require chemotherapy or radiotherapy in addition to surgery. However, most patients are very unfamiliar with and fearful of subsequent chemotherapy and radiotherapy; also, they may be concerned about the side effects of these treatments. Therefore, patients will want to understand their disease during treatment, strive for self-care, adapt to life changes as early as possible, and understand the disease response strategies appropriate to their situation [3]. Patients undergoing cancer treatment report high levels of physical care needs [34]. In the present study, the mHealth app intervention did reduce the physiological needs, psychological needs, communication needs, and care support needs of patients who received it more than the routine health care and instruction provided by nurse caregivers. However, compared to the control group, the health information needs in the intervention group did not improve (–3.29 vs –0.14). This result may have occurred because when patients found they could obtain more health information from the app, their demand for health information also grew. When refining the mHealth app, more questionnaires should be used to determine the needs of patients for other health information content, particularly at different points in disease progression.

Before using the mHealth app, the acceptance of such apps in the 2 groups of patients was low; however, the scores for the three acceptance variables (intention to use, perceived usefulness, and perceived ease of use) all increased significantly after 3 months of the mHealth app intervention. This result indicates that familiarity with the mHealth app reduced the uncertainty and increased the acceptance of using it. Similarly, other studies have found that well-designed smartphone apps help to enhance compliance with oral anticancer medications, even for patients who were not previously compliant [19,27,33]. A nurse-led prechemotherapy education intervention (ChemoEd) using DVDs for pretreatment consultation also demonstrated that a DVD-based intervention can significantly reduce the incidence and severity of sensory, psychological, and procedural concerns as well as of vomiting [14]. A review study also supported our findings that technology-based interventions can have positive effects on pain, depression, and quality of life in cancer patients [19]. The results of this and the above studies indicate that mHealth app interventions may also provide health education benefits for postoperative oral cancer patients.

### Limitations and Recommendations

The present study has several limitations. Due to time constraints, the number of trained researchers, budget constraints, and other factors, patients could only be followed for 3 months. Despite the positive results observed during this period, we recommend conducting longer intervention studies in the future, such as those including 3-month, 6-month, 9-month, and 12-month intervals. We believe that some statistically insignificant results will improve if the study is expanded to a longer time frame (eg, psychological needs and communication needs). In addition, all the oral cancer patients in this study were recruited from one medical center. Therefore, the results may not be applicable to oral cancer patients from other medical centers or patients treated at nonmedical centers. In the future, patients from different medical centers or nonmedical centers should be included. In addition, treatment plans for each patient can be included in the analysis to provide researchers with an understanding of the association of treatment plans with the care needs of postoperative patients. Based on participants’ feedback, rehabilitation videos and oral cancer support groups provided the most useful information, and the participants suggested that doctors and nursing staff should be invited to join the mHealth app to provide immediate consultation.

### Conclusions

An mHealth app intervention can significantly reduce physiological needs in postoperative oral cancer patients, and use of the mHealth app was highly accepted by patients. These data may also provide health care professionals with a better understanding of the optimal course of patient care after surgery. The main results of this study indicated that the mHealth app can be easily incorporated into routine care of postoperative oral cancer patients to conveniently provide medical information and improve patients’ self-management abilities, thereby reducing their physiological care needs and promoting better health.

## Appendix

Multimedia Appendix 1 Screenshots of the interfaces of the app.

Multimedia Appendix 2 Questionnaires used in the study.

Multimedia Appendix 3 CONSORT-EHEALTH checklist (V. 1. 6. 1).

## Abbreviations

CNQ-SF: short-form Cancer Needs Questionnaire
EORTC QLQ-C30: European Organization for Research and Treatment of Cancer Quality of Life Questionnaire Core 30
EORTC QLQ-H&N35: European Organization for Research and Treatment of Cancer Quality of Life Questionnaire Core 30 and Head and Neck Module
mHealth: mobile health
PDA: personal digital assistant
TAM: Technology Acceptance Module
WHO: World Health Organization
