# Supplementary figures and images for: Evaluating the Effects of a Mobile Health App on Reducing Patient Care Needs and Improving Quality of Life After Oral Cancer Surgery: Quasiexperimental Study
Source: JMIR Mhealth Uhealth. 2020 Jul 27;8(7):e18132. doi: 10.2196/18132 (PMC7418017; doi:10.2196/18132)

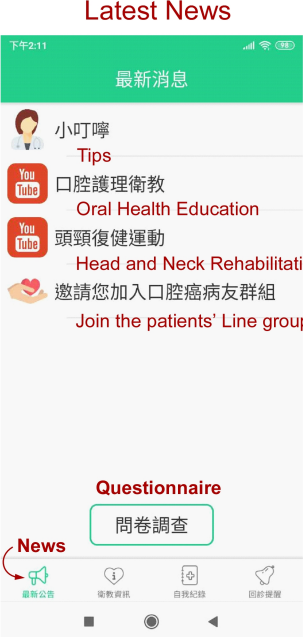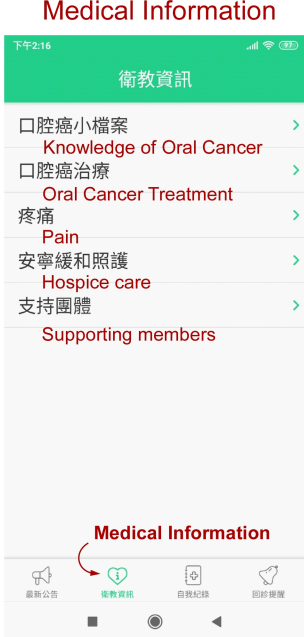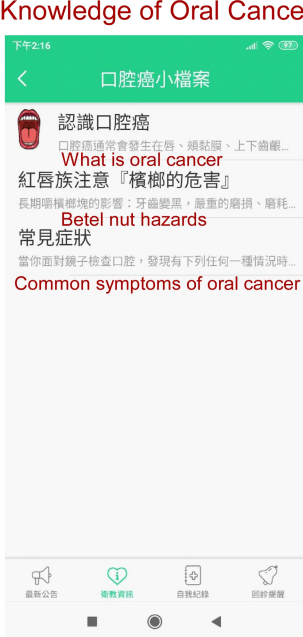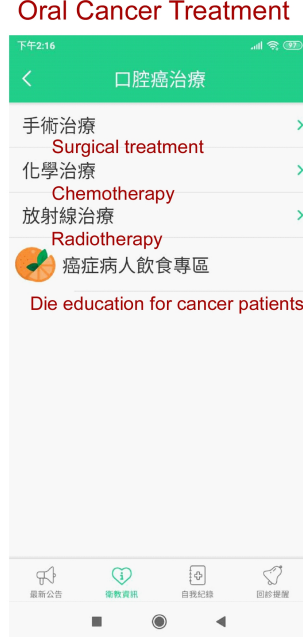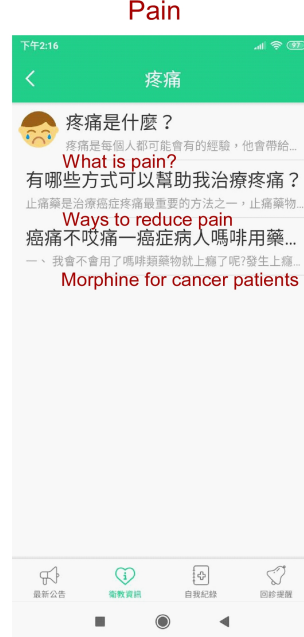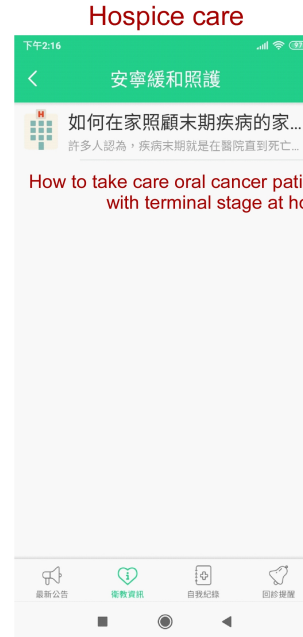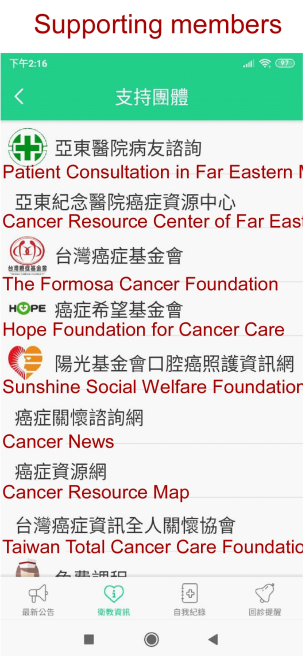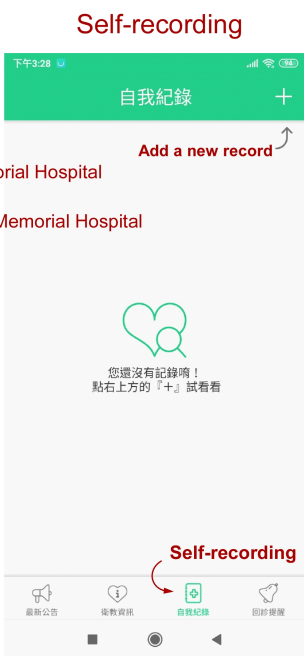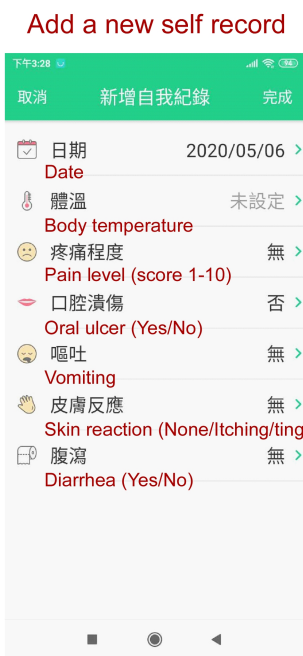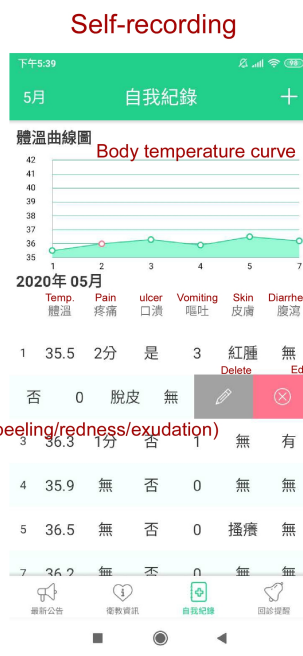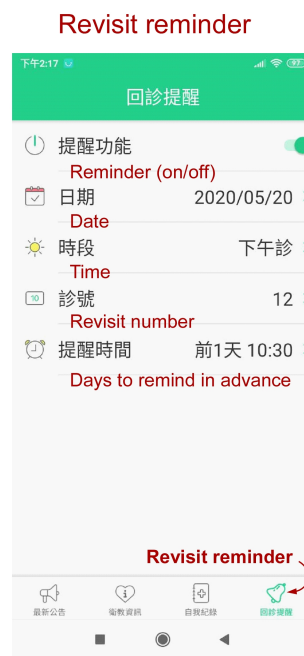

Supplement: Multimedia Appendix 1 [file mhealth_v8i7e18132_app1.pdf]
