# Supplementary material for: Evaluating the Effects of a Mobile Health App on Reducing Patient Care Needs and Improving Quality of Life After Oral Cancer Surgery: Quasiexperimental Study
Source: JMIR Mhealth Uhealth. 2020 Jul 27;8(7):e18132. doi: 10.2196/18132 (PMC7418017; doi:10.2196/18132)
Supplement: Multimedia Appendix 2 [file mhealth_v8i7e18132_app2.docx]

**癌症需求量表**

**Cancer Needs Questionnaire (CNQ) short-form**

請您以**手術後**這段時間的情況為準，依照您是否有下列情況：

如果沒有請圈選「不適用」；如果有某一項情況，但已得到適當的幫助，請圈選「已滿足」；如果有某一項情況是需要協助，請依照需要協助的程度圈選：「低度需要」、「中度需要」、「高度需要」。

Do you have the following conditions during the period after the surgery?

If there is no such situation, please circle "Not applicable". If you have certain situation, and it has been properly improved, please circle "Satisfied". If there is a situation that requires assistance, please circle the level of needs: "Low", "Moderate" or "High" need.

| 項目  Items | | 沒有需要  No need | | 有需要  Level of Needs | | |
| --- | --- | --- | --- | --- | --- | --- |
|  |  | 不適用  Not applicable | 已滿足  Satisfied | 低度  Low | 中度  Moderate | 高度  High |
| 1 | 需要處理精神不振和疲倦 | 1 | 2 | 3 | 4 | 5 |
|  | Dealing with lack of energy and tiredness |  |  |  |  |  |
| 2 | 需要克服睡眠狀況不良 | 1 | 2 | 3 | 4 | 5 |
|  | Coping with disturbed sleep |  |  |  |  |  |
| 3 | 需要持續處理家務 | 1 | 2 | 3 | 4 | 5 |
|  | Coping with keeping up with work around the home |  |  |  |  |  |
| 4 | 需要克服無法達成自己過去習慣作的事而帶來的挫敗 | 1 | 2 | 3 | 4 | 5 |
|  | Coping with frustration at not being able to do the things you used to |  |  |  |  |  |
| 5 | 需要克服害怕失去自己的獨立自主性 | 1 | 2 | 3 | 4 | 5 |
|  | Coping with fears about losing your independence |  |  |  |  |  |
| 6 | 需要克服心情煩悶和覺得一無是處 | 1 | 2 | 3 | 4 | 5 |
|  | Coping with feeling bored and/or useless |  |  |  |  |  |
| 7 | 需要處理心情的焦慮或壓力 | 1 | 2 | 3 | 4 | 5 |
|  | Dealing with anxiety or stress |  |  |  |  |  |
| 8 | 需要處理情緒低落或心情沮喪 | 1 | 2 | 3 | 4 | 5 |
|  | Dealing with feeling down or depressed |  |  |  |  |  |
| 9 | 需要處理癌症擴散或復發的恐懼 | 1 | 2 | 3 | 4 | 5 |
|  | Dealing with fears about the cancer spreading or returning |  |  |  |  |  |
| 10 | 需要處理可能會經驗到疼痛和痛苦的恐懼 | 1 | 2 | 3 | 4 | 5 |
|  | Coping with fears about the pain and suffering you might experience |  |  |  |  |  |
| 11 | 需要應付對接受癌症相關治療或手術的焦慮 | 1 | 2 | 3 | 4 | 5 |
|  | Coping with anxiety about having treatment or surgery |  |  |  |  |  |
| 12 | 需要應付害怕身體會失去功能或變壞 | 1 | 2 | 3 | 4 | 5 |
|  | Coping with fears about further physical disability or deterioration |  |  |  |  |  |
| 13 | 需要接受外表的變化和自我形象的變化 | 1 | 2 | 3 | 4 | 5 |
|  | Accepting the changes in your appearance and self-image |  |  |  |  |  |
| 14 | 需要應對擔心癌症是超出預期所能控制的 | 1 | 2 | 3 | 4 | 5 |
|  | Coping with worry that the cancer is beyond your control |  |  |  |  |  |
| 15 | 需要應付不確定的未來需要應付不確定的未來 | 1 | 2 | 3 | 4 | 5 |
|  | Coping with an uncertain future |  |  |  |  |  |
| 16 | 需要面對有關死亡和瀕臨死亡的感受 | 1 | 2 | 3 | 4 | 5 |
|  | Working through your feelings about death and dying |  |  |  |  |  |
| 17 | 需要學習去感受對自己狀況的控制 | 1 | 2 | 3 | 4 | 5 |
|  | Learning to feel in control of your situation |  |  |  |  |  |
| 18 | 需要應付與他人交談有關癌症病情的尷尬 | 1 | 2 | 3 | 4 | 5 |
|  | Coping with awkwardness in talking with others about the cancer |  |  |  |  |  |
| 19 | 需要調適面對他人對自己相處的態度和行為的改變 | 1 | 2 | 3 | 4 | 5 |
|  | Coping with changes in other people's attitudes and behavior towards you |  |  |  |  |  |
| 20 | 需要有家屬或朋友在醫院陪伴 | 1 | 2 | 3 | 4 | 5 |
|  | To be allowed to have family or friends with you in hospital |  |  |  |  |  |
| 21 | 當在門診或醫院時，需要讓您的隱私權受到更充分的保障 | 1 | 2 | 3 | 4 | 5 |
|  | To have your rights for privacy more fully protected when you are at the clinic or hospital |  |  |  |  |  |
| 22 | 需要醫護人員可以讓你放心，有關你的身體和情緒的反應是正常 | 1 | 2 | 3 | 4 | 5 |
|  | To be reassured by medical staff that your physical and emotional responses are normal |  |  |  |  |  |
| 23 | 在醫院中需要護理人員能迅速提供您身體方面的需求 | 1 | 2 | 3 | 4 | 5 |
|  | For nurses to attend promptly to your physical needs in hospital |  |  |  |  |  |
| 24 | 需要護理人員能瞭解並敏感地察覺您的感受和情緒需求 | 1 | 2 | 3 | 4 | 5 |
|  | For nurses to acknowledge, and show sensitivity to your feelings and emotional needs |  |  |  |  |  |
| 25 | 需要癌症專科治療醫師能瞭解並敏感地察覺您的感受和情緒需求 | 1 | 2 | 3 | 4 | 5 |
|  | For your cancer specialist to acknowledge and show sensitivity to your feelings and emotional needs |  |  |  |  |  |
| 26 | 需要得知所有要接受的每一項檢查和治療過程，都給予充分的解釋說明 | 1 | 2 | 3 | 4 | 5 |
|  | To be given a full explanation for every test and treatment procedure you go through |  |  |  |  |  |
| 27 | 在您同意接受治療或手術前，需要被充分告知優點和副作用 | 1 | 2 | 3 | 4 | 5 |
|  | To be fully informed about all of the benefits and side effects of treatment or surgery before you agree to have it |  |  |  |  |  |
| 28 | 需要被充分告知有關治療成功率的訊息 | 1 | 2 | 3 | 4 | 5 |
|  | To be fully informed about the odds of treatment success |  |  |  |  |  |
| 29 | 需要盡快被充分告知檢查結果 | 1 | 2 | 3 | 4 | 5 |
|  | To be fully informed about your test results as soon as possible |  |  |  |  |  |
| 30 | 需要充分了解有關的癌症可能影響生命的長度 | 1 | 2 | 3 | 4 | 5 |
|  | To be fully informed about the possible effects of the cancer on the length of your life |  |  |  |  |  |
| 31 | 需要被充分告知癌症緩解(減輕)的訊息 | 1 | 2 | 3 | 4 | 5 |
|  | To be fully informed about cancer remission |  |  |  |  |  |
| 32 | 需要被充分告知您能做什麼事來幫助自己復原 | 1 | 2 | 3 | 4 | 5 |
|  | To be fully informed about things you can do to help yourself get well |  |  |  |  |  |

**生活品質量表中文版**

**EORTC QLQ H&N35**

請您依照過去手術後一周內的生活經驗，依照自身感受程度進行勾選。分數愈低，表示症狀愈不明顯。

Please choose a suitable statement according to your feelings and life experience within one week after surgery. The lower the score, the less obvious the symptoms.

| 在過去一星期內（過去七天內）：  During the past week: | | 1 | 2 | 3 | 4 |
| --- | --- | --- | --- | --- | --- |
|  |  | 完全沒有 Not at all | 有一點 A little | 相當多 Quite a bit | 非常多 Very much |
| 1 | 您曾覺得口腔疼痛嗎？ | □ | □ | □ | □ |
|  | Have you had pain in your mouth |  |  |  |  |
| 2 | 您曾覺得下顎疼痛嗎？ | □ | □ | □ | □ |
|  | Have you had pain in your jaw |  |  |  |  |
| 3 | 您曾覺得口腔酸痛嗎？ | □ | □ | □ | □ |
|  | Have you had soreness in your mouth |  |  |  |  |
| 4 | 您曾覺得喉嚨痛嗎？ | □ | □ | □ | □ |
|  | Have you had a painful throat |  |  |  |  |
| 5 | 您吞嚥液體時曾有困難嗎？ | □ | □ | □ | □ |
|  | Have you had problems swallowing liquids |  |  |  |  |
| 6 | 您吞嚥濃湯時曾有困難嗎？ | □ | □ | □ | □ |
|  | Have you had problems swallowing pureed food |  |  |  |  |
| 7 | 您吞嚥固體食物時曾有困難嗎？ | □ | □ | □ | □ |
|  | Have you had problems swallowing solid food |  |  |  |  |
| 8 | 您吞嚥時曾經嗆到嗎？ | □ | □ | □ | □ |
|  | Have you choked when swallowing |  |  |  |  |
| 9 | 您曾有牙齒方面的問題嗎？ | □ | □ | □ | □ |
|  | Have you had problems with your teeth |  |  |  |  |
| 10 | 您張大嘴巴曾有困難嗎？ | □ | □ | □ | □ |
|  | Have you had problems opening your mouth wide |  |  |  |  |
| 11 | 您曾覺得嘴巴乾乾的嗎？ | □ | □ | □ | □ |
|  | Have you had a dry mouth |  |  |  |  |
| 12 | 您曾覺得唾液黏稠嗎？ | □ | □ | □ | □ |
|  | Have you had sticky saliva |  |  |  |  |
| 13 | 您曾有嗅覺方面的問題嗎？ | □ | □ | □ | □ |
|  | Have you had problems with your sense of smell |  |  |  |  |
| 14 | 您曾有味覺方面的問題嗎？ | □ | □ | □ | □ |
|  | Have you had problems with your sense of taste |  |  |  |  |
| 15 | 您曾有咳嗽嗎？ | □ | □ | □ | □ |
|  | Have you coughed |  |  |  |  |
| 16 | 您曾有聲音沙啞嗎？ | □ | □ | □ | □ |
|  | Have you been hoarse |  |  |  |  |
| 17 | 您是否曾覺得不舒服？ | □ | □ | □ | □ |
|  | Have you felt ill |  |  |  |  |
| 18 | 您曾為自己的外觀感到困擾嗎？ | □ | □ | □ | □ |
|  | Has your appearance bothered you |  |  |  |  |
| 19 | 您進食曾感到困擾嗎？ | □ | □ | □ | □ |
|  | Have you had trouble eating |  |  |  |  |
| 20 | 您在家人面前進食曾感到困擾嗎？ | □ | □ | □ | □ |
|  | Have you had trouble eating in front of your family |  |  |  |  |
| 21 | 您在其他人面前進食曾感到困擾嗎？ | □ | □ | □ | □ |
|  | Have you had trouble eating in front of other people |  |  |  |  |
| 22 | 您是否曾覺得難以享受進餐的樂趣？ | □ | □ | □ | □ |
|  | Have you had trouble enjoying your meals |  |  |  |  |
| 23 | 您與別人交談曾感到困擾嗎？ | □ | □ | □ | □ |
|  | Have you had trouble talking to other people |  |  |  |  |
| 24 | 您用電話交談曾感到困擾嗎？ | □ | □ | □ | □ |
|  | Have you had trouble talking on the telephone |  |  |  |  |
| 25 | 您和家人交往接觸曾感到困擾嗎？ | □ | □ | □ | □ |
|  | Have you had trouble having social contact with your family | |  |  |  |
| 26 | 您和朋友交往接觸曾感到困擾嗎？ | □ | □ | □ | □ |
|  | Have you had trouble having social contact with friends |  |  |  |  |
| 27 | 您外出至公共場合曾感到困擾嗎？ | □ | □ | □ | □ |
|  | Have you had trouble going out in public |  |  |  |  |
| 28 | 您和家人或朋友有身體接觸時，曾感到困 | □ | □ | □ | □ |
|  | Have you had trouble having physical |  |  |  |  |
| 29 | 您是否對「性」曾感到比較沒有興趣？ | □ | □ | □ | □ |
|  | Have you felt less interest in sex |  |  |  |  |
| 30 | 您是否曾感到比較無法享受性生活？ | □ | □ | □ | □ |
|  | Have you felt less sexual enjoyment |  |  |  |  |
|  |  |  |  |  |  |
|  | 在過去一星期內（過去七天內）：  During the past week: | 沒有 No | 有 Yes |  |  |
| 31 | 您曾使用止痛藥嗎？ | □ | □ |  |  |
|  | Have you used pain-killers |  |  |  |  |
| 32 | 您曾使用任何營養補充品嗎？ | □ | □ |  |  |
|  | Have you taken any nutritional supplements (excluding vitamins) | |  |  |  |
| 33 | 您曾使用餵食管進食嗎？ | □ | □ |  |  |
|  | Have you used a feeding tube |  |  |  |  |
| 34 | 您曾有體重減輕嗎？ | □ | □ |  |  |
|  | Have you lost weight |  |  |  |  |
| 35 | 您曾有體重增加嗎？ | □ | □ |  |  |
|  | Have you gained weight |  |  |  |  |

**癌症病人生活品質量表**

**EORTC QLQ-C30**

|  | 題目  Item | | | | | 1 | 2 | 3 | | 4 |
| --- | --- | --- | --- | --- | --- | --- | --- | --- | --- | --- |
|  |  |  |  |  |  | 完全沒有  Not at all | 有一點  A little | 相當多  Quite a bit | | 非常多  Very much |
| 1 | 您從事一些費力的活動，如攜帶重的購物袋 | | | | | □ | □ | □ | | □ |
|  | Do you have any trouble doing strenuous activities, like carrying a heavy shopping bag or a suitcase? | | | | |  |  |  | |  |
| 2 | 您從事長距離步行，是否有困難？ | | | | | □ | □ | □ | | □ |
|  | Do you have any trouble taking a long walk? | | | | |  |  |  | |  |
| 3 | 您在戶外從事短距離步行，是否有困難？ | | | | | □ | □ | □ | | □ |
|  | Do you have any trouble taking a short walk outside of the house? | | | | |  |  |  | |  |
| 4 | 您在白天是否需要待在床上或椅子上？ | | | | | □ | □ | □ | | □ |
|  | Do you need to stay in bed or a chair during the day? | | | | |  |  |  | |  |
| 5 | 您進食、穿衣、洗澡或上廁所需要別人幫助嗎？ | | | | | □ | □ | □ | | □ |
|  | Do you need help with eating, dressing, washing yourself or using the toilet? | | | | |  |  |  | |  |
|  |  | | | | |  |  |  | |  |
|  | 在過去一個星期內(過去七天內) | | | | |  |  |  | |  |
|  | During the past week: | | | | |  |  |  | |  |
| 6 | 您在從事工作或日常活動上是否受到限制？ | | | | | □ | □ | □ | | □ |
|  | Were you limited in doing either your work or other daily activities? | | | | |  |  |  | |  |
| 7 | 您在從事嗜好或休閒活動上是否受到限制？ | | | | | □ | □ | □ | | □ |
|  | Were you limited in pursuing your hobbies or other leisure time activities? | | | | |  |  |  | |  |
| 8 | 您呼吸會喘嗎？ | | | | | □ | □ | □ | | □ |
|  | Were you short of breath? | | | | |  |  |  | |  |
| 9 | 您曾感到疼痛嗎？ | | | | | □ | □ | □ | | □ |
|  | Have you had pain? | | | | |  |  |  | |  |
| 10 | 您需要休息嗎？ | | | | | □ | □ | □ | | □ |
|  | Did you need to rest? | | | | |  |  |  | |  |
| 11 | 您曾難於入睡嗎？ | | | | | □ | □ | □ | | □ |
|  | Have you had trouble sleeping? | | | | |  |  |  | |  |
| 12 | 您曾感到虛弱嗎？ | | | | | □ | □ | □ | | □ |
|  | Have you felt weak? | | | | |  |  |  | |  |
| 13 | 您曾缺乏食欲嗎？ | | | | | □ | □ | □ | | □ |
|  | Have you lacked appetite? | | | | |  |  |  | |  |
| 14 | 您曾感到噁心嗎？ | | | | | □ | □ | □ | | □ |
|  | Have you felt nauseated? | | | | |  |  |  | |  |
| 15 | 您曾嘔吐嗎？ | | | | | □ | □ | □ | | □ |
|  | Have you vomited? | | | | |  |  |  | |  |
| 16 | 您曾便祕嗎？ | | | | | □ | □ | □ | | □ |
|  | Have you been constipated? | | | | |  |  |  | |  |
| 17 | 您曾腹瀉嗎？ | | | | | □ | □ | □ | | □ |
|  | Have you had diarrhea? | | | | |  |  |  | |  |
| 18 | 您疲倦嗎？ | | | | | □ | □ | □ | | □ |
|  | Were you tired? | | | | |  |  |  | |  |
| 19 | 疼痛干擾您的日常活動嗎？ | | | | | □ | □ | □ | | □ |
|  | Did pain interfere with your daily activities? | | | | |  |  |  | |  |
| 20 | 您曾否難注意力集中在一些事情上，如看報紙或看電視？ | | | | | □ | □ | □ | | □ |
|  | Have you had difficulty in concentrating on things, like reading a newspaper or watching television? | | | | |  |  |  | |  |
| 21 | 您覺得緊張嗎？ | | | | | □ | □ | □ | | □ |
|  | Did you feel tense? | | | | |  |  |  | |  |
| 22 | 您感到憂鬱嗎？ | | | | | □ | □ | □ | | □ |
|  | Did you worry? | | | | |  |  |  | |  |
| 23 | 您覺得容易發怒嗎？ | | | | | □ | □ | □ | | □ |
|  | Did you feel irritable? | | | | |  |  |  | |  |
| 24 | 您覺得情緒低落嗎？ | | | | | □ | □ | □ | | □ |
|  | Did you feel depressed? | | | | |  |  |  | |  |
| 25 | 您曾感到記憶困難嗎？ | | | | | □ | □ | □ | | □ |
|  | Have you had difficulty remembering things? | | | | |  |  |  | |  |
| 26 | 您的身體狀況或醫療過程是否曾干擾您的家庭生活？ | | | | | □ | □ | □ | | □ |
|  | Has your physical condition or medical treatment interfered with your family life? | | | | |  |  |  | |  |
| 27 | 您的身體狀況或醫療過程是否曾干擾您的社交活動？ | | | | | □ | □ | □ | | □ |
|  | Has your physical condition or medical treatment interfered with your social activities? | | | | |  |  |  | |  |
| 28 | 您的身體狀況或醫療過程是否曾造成您財務上的困難？ | | | | | □ | □ | □ | | □ |
|  | Has your physical condition or medical treatment caused you financial difficulties? | | | | |  |  |  | |  |
|  |  | | | | |  |  |  | |  |
|  | 以下問題，請在1到7之間 圈選最適合您的答案 | | | | | | | | | |
|  | For the following questions, please circle the number between 1 and 7 that best applies to you | | | | | | | | | |
| 29 | 您如何評定過去一星期內(過去七天內)您整體的健康？ | | | | |  |  |  | |  |
|  | How would you rate your overall health during the past week? | | | | |  |  |  | |  |
|  | 非常差  Very poor |  |  |  |  | |  | | 極好  Excellent | |
|  | 1 | 2 | 3 | 4 | 5 | | 6 | | 7 | |
| 29 | 您如何評定過去一星期內(過去七天內)您整體的生活品質？ | | | | | |  |  | |  |
|  | How would you rate your overall quality of life during the past week? | | | | | |  |  | |  |
|  | 非常差  Very poor |  |  |  |  | |  | | 極好  Excellent | |
|  | 1 | 2 | 3 | 4 | 5 | | 6 | | 7 | |

**科技接受模式**

**Technology Acceptance Model (TAM)**

1. 當您遇到對於疾病或治療照護不了解時，您是如何解決？（可複選）

How do you solve the questions when you don’t know about the disease or nursing care?

□上網查 (Check Online)　□ 下次回診問醫師 (Ask doctors in revisit)

□打電話問護理人員 (Call nursing staff)　□其他 (Others)

1. 是否曾接受醫護人員照護方面衛教?

Have you ever received health education from medical staff?

□ 是 (Yes)：

□大部分都記得 (Remember most of the content)

□部分記得 (Remember part of the content)

□大都忘記 (Most of the content is forgotten)

□ 否 (No)

若您有機會使用口腔癌照護APP，您認為：（前測）

If you have the opportunity to use the oral cancer APP…… (test before download and use the mHealth APP)

|  | 題目 | | 非常  不同意 | 不同意 | 普通 | 同意 | 非常  同意 |
| --- | --- | --- | --- | --- | --- | --- | --- |
|  | Item | | Strongly Disagree | Disagree | Somewhat agree | Agree | Strongly Agree |
| 使用意願 Intention to Use | 1 | 假如有口腔癌照護APP，我會願意下載。 | □ | □ | □ | □ | □ |
|  |  | If there is an oral cancer care APP, I am willing to download it. |  |  |  |  |  |
|  | 2 | 假如我有現在有安裝口腔癌照護APP，我會去使用。 | □ | □ | □ | □ | □ |
|  |  | If I have installed the oral cancer care APP, I will use it. |  |  |  |  |  |
|  | 3 | 假如我有安裝口腔癌照護APP，在未來一個月內，我將會去使用此系統。 | □ | □ | □ | □ | □ |
|  |  | If I have installed the oral cancer care APP, I will use it within next month. |  |  |  |  |  |
| 有用性 Perceived usefulness | 4 | 假如我有安裝口腔癌照護APP，我覺得可以更快速獲得資訊。 | □ | □ | □ | □ | □ |
|  |  | If I have installed the oral cancer care APP, I think I can obtain information quickly. |  |  |  |  |  |
|  | 5 | 假如我有安裝口腔癌照護APP，我覺得可以獲得更完整的資訊內容。 | □ | □ | □ | □ | □ |
|  |  | If I have installed the oral cancer care APP, I think I can obtain more complete information. |  |  |  |  |  |
| 易用性 Perceived Ease of Use | 6 | 假如我有安裝口腔癌照護APP，我覺得APP健康資訊系統的介面可以很清楚且容易理解。 | □ | □ | □ | □ | □ |
|  |  | If I have installed the oral cancer care APP, I think that the interface of Health Information System in this APP can be very clear and easy to understand. |  |  |  |  |  |
|  | 7 | 假如我有安裝口腔癌照護APP，我覺得我很容易就可以上手。 | □ | □ | □ | □ | □ |
|  |  | If I have installed the oral cancer care APP, I think I can easily to get started. |  |  |  |  |  |
|  | 8 | 假如我有安裝口腔癌照護APP我覺得APP系統可能會比紙本單張更容易保存資料 | □ | □ | □ | □ | □ |
|  |  | If I have installed the oral cancer care APP, I think the APP system may be easier to save information than the paper records. |  |  |  |  |  |

您對於APP照護系統使用建議（後測）：

Recommendations after using the mHealth APP care system (test only after use)

|  | 題目 | | 非常  不同意 | 不同意 | 普通 | 同意 | 非常  同意 |
| --- | --- | --- | --- | --- | --- | --- | --- |
|  | Item | | Strongly Disagree | Disagree | Somewhat agree | Agree | Strongly Agree |
| 使用意願 Intention to Use | 1 | 假如我有機會使用口腔癌照護APP，我有意願使用。 | □ | □ | □ | □ | □ |
|  |  | If I have the opportunity to use the oral cancer care APP, I am willing to use it. |  |  |  |  |  |
|  | 2 | 我很樂意繼續使用口腔癌照護APP。 | □ | □ | □ | □ | □ |
|  |  | I am happy to continue using the oral cancer care APP. |  |  |  |  |  |
|  | 3 | 未來一個月內，我將會使用口腔癌照護APP。 | □ | □ | □ | □ | □ |
|  |  | In the next month, I will use the oral cancer care APP. |  |  |  |  |  |
| 有用性 Perceived usefulness | 4 | 使用口腔癌照護APP讓我更快速獲得資訊。 | □ | □ | □ | □ | □ |
|  |  | Using the oral cancer care APP allows me to get information more quickly. |  |  |  |  |  |
|  | 5 | 使用口腔癌照護APP讓我獲得更完整的資訊內容。 | □ | □ | □ | □ | □ |
|  |  | Using the oral cancer care APP allows me to obtain more complete information. |  |  |  |  |  |
| 易用性 Perceived Ease of Use | 6 | 我覺得口腔癌照護APP的介面是清楚且容易理解。 | □ | □ | □ | □ | □ |
|  |  | I think the interface of the oral cancer care APP is clear and easy to understand. |  |  |  |  |  |
|  | 7 | 我認為口腔癌照護APP操作很容易上手。 | □ | □ | □ | □ | □ |
|  |  | I think this operation of the oral cancer care APP is easy to get started. |  |  |  |  |  |
|  | 8 | 我認為口腔癌照護APP比紙本單張更容易保存資料 | □ | □ | □ | □ | □ |
|  |  | I think that this oral cancer care APP is easier to save information than paper records. |  |  |  |  |  |
| 態度 Attitude | 9 | 我喜歡使用口腔癌照護APP健康資訊系統。 | □ | □ | □ | □ | □ |
|  |  | I like to use this oral cancer health care APP to obtain health information. |  |  |  |  |  |
|  | 10 | 對於使用口腔癌照護APP健康資訊系統我感到很棒 | □ | □ | □ | □ | □ |
|  |  | I feel great about using the oral cancer care APP health information system. |  |  |  |  |  |
| 意圖 Intention | 11 | 我認為口腔癌照護APP是值得使用。 | □ | □ | □ | □ | □ |
|  |  | I think the oral cancer care APP is worth using. |  |  |  |  |  |
|  | 12 | 未來我會經常使用口腔癌照護APP。 | □ | □ | □ | □ | □ |
|  |  | In the future, I will often use this oral cancer care APP. |  |  |  |  |  |
|  | 13 | 口腔癌照護APP能滿足我健康訊息需求 | □ | □ | □ | □ | □ |
|  |  | The oral cancer care APP can satisfy my needs for health information. |  |  |  |  |  |
|  | 14 | 口腔癌照護APP對我有實質的幫助 | □ | □ | □ | □ | □ |
|  |  | The oral cancer care APP is actually helpful for me. |  |  |  |  |  |
|  | 15 | 使用APP可提升我對照護上的信心 | □ | □ | □ | □ | □ |
|  |  | Using this oral health APP can improve my confidence in nursing care. |  |  |  |  |  |

您對於口腔癌APP滿意程度如何?

How satisfied are you with the oral cancer app?

|  | 題目 | | 非常  不滿意 | 不滿意 | 普通 | 滿意 | 非常  滿意 |
| --- | --- | --- | --- | --- | --- | --- | --- |
|  | Item | | Extremely Dissatisfied | Dissatisfied | Somewhat agree | Satisfied | Very Satisfied |
| 滿意度 Satisfaction | 1 | 口腔癌簡介 | □ | □ | □ | □ | □ |
|  |  | Introduction of oral cancer |  |  |  |  |  |
|  | 2 | 口腔癌手術 | □ | □ | □ | □ | □ |
|  |  | Surgery of oral cancer |  |  |  |  |  |
|  | 3 | 化學治療照護 | □ | □ | □ | □ | □ |
|  |  | Chemotherapy care |  |  |  |  |  |
|  | 4 | 放射線治療照護 | □ | □ | □ | □ | □ |
|  |  | Radiotherapy care |  |  |  |  |  |
|  | 5 | 復健影片 | □ | □ | □ | □ | □ |
|  |  | Rehabilitation video |  |  |  |  |  |
|  | 6 | 自我紀錄功能 | □ | □ | □ | □ | □ |
|  |  | Self-recording |  |  |  |  |  |
|  | 7 | 回診提醒功能 | □ | □ | □ | □ | □ |
|  |  | Revisit reminder |  |  |  |  |  |
|  | 8 | 支持團體連結 | □ | □ | □ | □ | □ |
|  |  | Link to supporting groups/members |  |  |  |  |  |
|  | 9 | APP介面 | □ | □ | □ | □ | □ |
|  |  | APP interface |  |  |  |  |  |
|  | 10 | 您對口腔癌APP的使用建議:_________________ | | | | | |
|  |  | Your suggestions for the use of oral cancer APP: _________________ | | | | | |
